# Analysis of the proportion of university teaching hospital gastric cancer data included in the Zambia national cancer registry

**DOI:** 10.4314/ahs.v23i1.46

**Published:** 2023-03

**Authors:** Samson Shumba, Violet Kayamba

**Affiliations:** 1 University of Zambia School of Public Health, Department of Epidemiology and Biostatistics, Lusaka, Zambia; 2 Tropical Gastroenterology and Nutrition Group, University of Zambia School of Medicine, PO Box 50398, Lusaka, Zambia

**Keywords:** Gastric cancer, survival, Zambia, outcomes

## Abstract

**Background:**

The exact prevalence of gastric cancer in Zambia is unknown. The aim of this study was to determine the proportion of gastric cancer cases seen at the largest hospital in Zambia, whose records were included in the Zambia National Cancer Registry (ZNCR).

**Methods:**

We reviewed gastric cancer records between January 2015 and December 2017 from the University Teaching Hospital, Cancer Diseases Hospital and ZNCR. Statistical analysis was done using Stata version 15.

**Results:**

We reviewed 94 patient records (42 were females) with a mean age of 59 years (standard deviation, 14.9). Histologically, the majority of the cases were adenocarcinoma (84%). Of the reviewed records, only 39/94 (42%) had their data included in the ZNCR. Median time to from endoscopic diagnosis to histopathological confirmation was 11 days (inter-quartile range; 7-20). Median time from histological confirmation to the Cancer Diseases Hospital for therapy was 8 days (IQR 2-19). Overall median survival time for gastric cancer patients was 290 days (CI 95%, 112 – 1225).

**Conclusions:**

Data on gastric cancer reported in the ZNCR are an underestimate of the true disease burden and there is need to strengthen data collection strategies for gastric cancer in Zambia.

## Background

Gastric cancer is a malignant tumour that can arise from any part of the stomach, including the cardia, fundus, body and antrum. Globally, It is the fifth most common cancer and the fourth most common cause of cancer related deaths.^1^ In 2020, there were over a million new cases of gastric cancer and more than 700,000 related deaths. 1 In Africa, gastric cancer ranks ninth among new cases and it is the seventh leading cause of cancer related deaths.^2^ Projected figures show that there will be an estimated 97% increase of gastric cancer cases in Africa by 2040, the largest proportion globally.^2^ However, with a paucity of population-based cancer registries on the continent, data from many of the African countries are estimates. Of the 13,831 cancer cases reported from Zambia in 2020, 285 (2.1%) were gastric cancer, making it the eight most common cancer in the country.^2^ The estimated age standardised incidence rate for gastric cancer in Zambia is 4.0 per 100,000 while that for females is 3.7 per 100,000 with an overall mortality of 3.4 per 100,000.^2^

In many parts of sub-Saharan Africa, Zambia included, medical research has largely focussed on communicable diseases with relatively lower published data on non-communicable diseases such as gastric cancer. The true burden of gastric cancer is therefore not very clear as population-based cancer registries are scarce in Africa.^3^,^4^,^5^ Cancer cases are under-reported due to lack of good data management system to be able to follow up the cases and limited diagnostic facilities. However, in the recent years, there has been a steady increase in the number of gastric cancer related publications. From some of these works, it was reported that gastric cancer occurrence in Zambia was not associated with the Human Immunodeficiency Virus (HIV) infection,^6^ had a high occurrence among young adults,^6^ had poor patient outcomes^7^ and was probably driven by long-term exposure to biomass smoke.^8^ It was also reported that there was a high proportion of microsatellite unstable gastric cancer cases in Zambia.^9^ None of these studies were population based and the exact country wide prevalence of gastric cancer remains unknown.

Individuals presenting with suspected gastric cancer in Zambia are generally first evaluated at various public and private health institutions around the country. If available, endoscopic services are provided. Endoscopic biopsies from suspicious lesions are sent for histological diagnosis and upon histological confirmation of the cancer, patients are referred to the Cancer Diseases Hospital (CDH), the sole institution with comprehensive cancer treatment capabilities in Zambia. Records from the CDH are then directly recorded into the Zambia National Cancer Registry (ZNCR). There is also provision for the ZNCR to obtain cancer related data directly from individual health facilities.

In this study, we set out to estimate the proportion of gastric cancer cases diagnosed at the University Teaching Hospital (UTH), whose records are included in the ZNCR, which is the national database recording cancer cases.

## Methods

Data from this study were obtained from the UTH, CDH and the ZNCR in Lusaka Zambia. Within the Zambian referral system, patients are first seen in primary and secondary health centres and if deemed necessary, they are referred to tertiary centres such as the UTH. UTH is the largest referral hospital in Zambia, attending to patients from all ten provinces of the country. If gastric cancer is suspected, biopsies are taken during endoscopy and examined histologically within UTH histopathology laboratory. Confirmed cases are then referred to the CDH for treatment. Data from CDH are directly fed onto the ZNCR database, where the final information on the occurrence of these cancers are collected and stored. There is no delay in this process as it is done electronically. These services are generally free, but specific investigations attract a minimal, highly subsidised fee. There is a social welfare system providing assistance to patients who cannot afford these fees.

All records on patients suspected to have a primary diagnosis of gastric cancer were retrieved from the UTH endoscopy unit and histological confirmation sought from the histopathology laboratory covering 1^st^ January 2015 to 31^st^ December 2017. This was consecutive sampling. Only those with histologically confirmed gastric cancer were included in the final analysis. Patient names and in some cases hospital numbers were used to identify cases and facilitate accurate follow-up of individual records. We then compared these records with the CDH and ZNCR databases to determine the proportion of patients with confirmed gastric cancer that were included in the ZNCR. Analysis of the retrieved records was done in March, 2020. The University of Zambia Biomedical Research Ethics Committee, ref number 381-2019 and National Health Research Authority approved this study.

## Data Analysis

Frequencies and percentages were used to describe categorical variables. For continuous variables, means with standard deviations (SD) and medians with interquartile ranges (IQR) were reported after checking for normality with the Shapiro Wilk test. Where appropriate a t-test, rank sum or Kruskal Wallis were used to check for associations involving continuous variables. For categorical variables, Chi-square or Fisher's exact tests ere used depending on the number of cases evaluated. Survival rates were computed using Kaplan-Meier graphs. Stata version 15 was used for the analysis.

## Results

**Figure 1:**
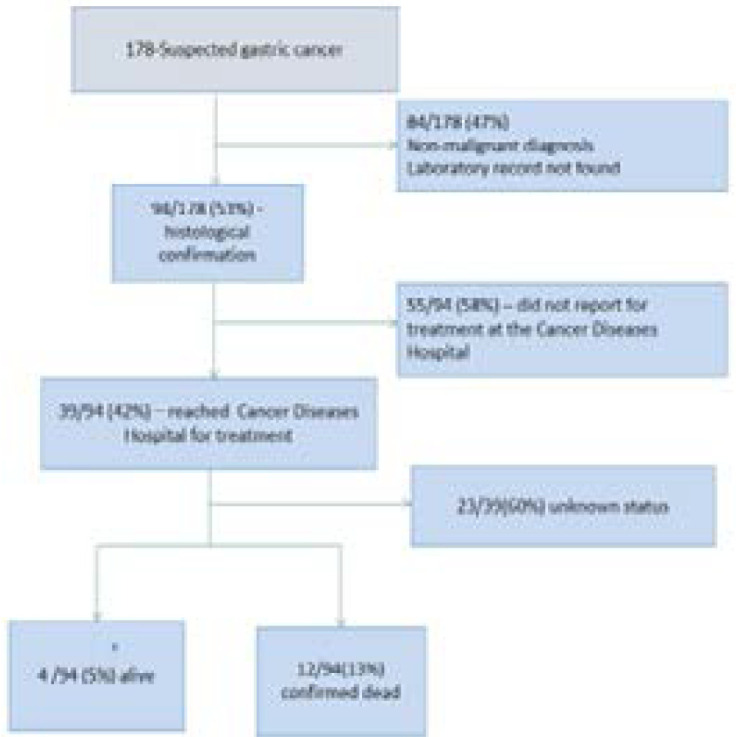
Flow chart showing patient records that were included in the study

Records for 178 patients suspected with gastric cancer were retrieved form the UTH endoscopy unit. Histological confirmation for gastric cancer was available for 94/178 (53%) of these suspected cases and these were included in the subsequent analysis and 84% of these were adenocarcinomas and the remaining were squamous cell carcinoma (6%), Kaposi's sarcoma (6%), neuroendocrine malignancy (1%) or metastatic lesions (3%). Of these, 42 were females. Age ranged from 30 to 93 years with a mean of 59 (SD=14.9) years and median body mass index (BMI) of 17.7Kg/m^2^ (IQR 15.6 -19.5 Kg/m^2^). Other basic characteristics were as outlined in Table 1. Review of the records at the Cancer Diseases Hospital (CDH) revealed that only 39/94 (42%) of the patients with confirmed gastric cancer reported for therapy. While 55/94 (58%) were lost to follow up, Figure 1. At the time of data collection, 4/94 (5%) of the patients were still in active care at CDH with 12/94 (13%) confirmed dead, Figure 1. The median time from endoscopic diagnosis to histopathological confirmation was 11 days (IQR 7-20) days. It took a median time of 8 days (IQR 2-19) days from histological confirmation of gastric cancer to arrival at CDH for initial treatment.

**Table 1:** Baseline characteristics of patients and cancers included in the study

Characteristics	Gastric Cancer N (%) n=94
Age; mean (SD), years	58.5 (15)
Sex: Female Male Missing	42 (45) 49 (52) 3 (3)
Major clinical symptom: Pain Haematemesis Persistent vomiting Dysphagia Microcytic anaemia Other	16 (17) 17 (18) 13 (14) 20 (21) 6 (6) 22 (24)
Occupation: Unemployed Employed Missing	18 (19) 15 (16) 61 (65)
Time to Diagnosis in days; median (IQR); n=94	11 (7–20)
Time to Cancer Diseases Hospital in days; median (IQR); n=94	16 (10–38)
BMI; median (IQR) kg/m^2^; n=94	17.7 (15.6–19.5)
Distance from residence to hospital; median (IQR) Km; n=94	17.9 (183.5)
Tumour grade: Poorly Differentiated Moderately Differentiated Well Differentiated Missing	12 (13) 19 (20) 7 (7) 56 (59)
HIV Status: HIV Negative HIV Positive Unknown	11 (11) 3 (3) 80 (84)
Treatment administered: n=16 Chemotherapy Radiation Chemotherapy and Radiation	7 (44) 7 (44) 2 (13)

*Included under other symptoms are melaena, odynophagia and abdominal mass

**Figure 2:**
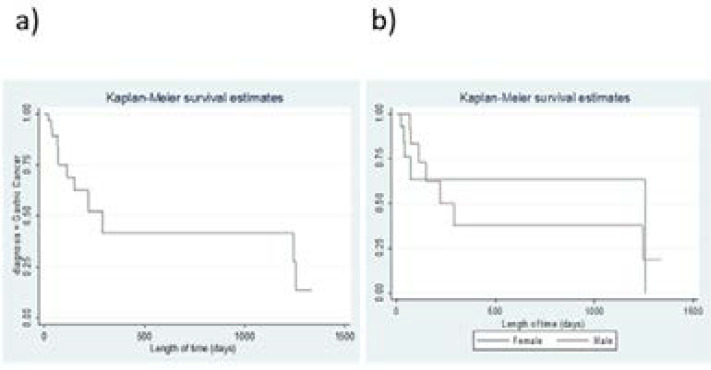
Kaplan-Meier curves showing survival for histologically confirmed gastric cancer (a) Overall survival, (b)survival by sex

Overall median time to gastric cancer confirmation was 290 (CI 95%; 112 – 1225) days (using Kaplan Meier survival estimates), Figure 2. Median survival time for adenocarcinoma was 1245 days, 290 days for squamous cell carcinoma and 35 days for metastatic disease. These differences were not statistically significant (log rank test; p=0.05). There was no association between sex and survival; females (1255 days) and males (220 days; log rank test, p=0.60).

## Discussion

In this retrospective audit, we report that less than half of patients with histologically confirmed gastric cancer were seen at the Cancer Diseases Hospital and their cases reported in the Zambia National Cancer Registry. Our findings are an illustration of the proportion of gastric cancer cases that are included in the national database from which estimates for the burden of the disease in Zambia are drawn.

We previously reported that delays in referral for endoscopy was one of the major contributors to late gastric cancer diagnosis.^10^ In that study, we found that within two weeks of first noticing symptoms, patients did seek medical attention, but it took another three months before they were subsequently referred for endoscopy, during which time their disease condition was worsening.^10^ It is therefore, important to identify gaps within the health care system that are contributing to delayed service provision.

In this current audit, the proportion of individuals lost to follow up after endoscopy diagnosis is alarming. These individuals were therefore not included in the ZNCR data base. There could be several reasons why these patients never returned. Gastric cancer does not just ‘disappear’ and it is unlikely that patients simply decided to stay in their homes and ignore their illnesses. It is therefore, logical to assume that many of these individuals died, providing more evidence of advanced disease at first presentation for endoscopy. On the other hand, there are some social and economic barriers that could have prevented these patients from returning to hospital. These could have included the following; cost of transportation, traditional beliefs, poor understanding of the diagnosed disease or unclear instructions by the healthcare providers. The cost of investigations and medication could have deterred some patients, but this could not have been the predominant factor as mechanisms for assistance in such situations are available.

It is clear that data collection on gastric cancer needs to be improved in Zambia. The burden of the disease is much higher that is currently reported. This has an impact on the importance that is attached to this disease condition. In addition, as many of the patients do not reach the CDH, it is difficult to ascertain treatment outcomes. We previously published data showing a very low survival rate for gastric cancer patients in Zambia 7 and these rates are similarly low in other African^11^ and more in developed countries as well.^13^ Like many other cancers, gastric cancer survival largely depends on the stage at which it is diagnosed.^14^

One limitation of this study is that it was hospital based and some of the records were incomplete. There are some variables that were not complete such as the grading of the tumours. In such cases, we could have misclassified some effects. Patient outcomes could have been affected by tumour staging, consistency or adherence to therapy, and coexisting conditions. We did not have these details. In addition, it was conducted at a single centre and therefore these results might not necessarily be generalizable to the whole country. However, UTH is the largest referral hospital and we were able to demonstrate gaps gastric cancer record capturing. We have clearly shown the need to collect cancer data directly from the health centres in order to understand the true epidemiology of gastric cancer whose occurrence is increasing in Zambia.^15^

With these findings, we recommend that better cancer data collection strategies be implemented in Zambia. Data on cancer diagnosis should be collected at first contact with the patients and not only after they report to CDH for treatment.

## Conclusion

The reported prevalence of gastric cancer in Zambia is an underestimate as many of the cases are not included in the database. There is need to improve data collection by the ZNCR from healthcare centres.
